# Seeking truth: Less about being right, more about being less wrong

**DOI:** 10.1016/j.ccrj.2025.100144

**Published:** 2025-10-14

**Authors:** Derek C. Angus

**Affiliations:** University of Pittsburgh School of Medicine, 614 Scaife Hall, Pittsburgh, PA 15232, USA

Many beautiful words have been written about Rinaldo in the last few months, capturing his boundless passion and enthusiasm, his incisive and inquisitive mind, and his warm, caring, and funny personality. Reading them broke my heart. These qualities doubtless all contributed greatly to the stature he achieved worldwide. They also drive the huge sense of loss so many of us feel, both for his family and dearest friends and for ourselves as a global community. It is hard to even imagine a world without him. He leaves behind a truly remarkable scientific legacy. By all accounts, Australia’s most prolific scientist, he wrote over 2000 publications and was cited >200,000 times, in collaboration with thousands of coauthors from all over the globe.^1^

In writing this piece, I found myself thinking about what it was that made Rinaldo such a remarkable clinician-scientist. I concluded that it has something to do with the way he sought truth. He seemed to appreciate we often know far less than we realise, wallowing in ignorance and uncertainty and that the path forward would rarely be lit by bright lucid truths and clarity. Rather, we make progress by stumbling forward with small, often unexpected, insights. We should not be so arrogant as to assume we are right but take comfort simply in being less wrong.

I first met Rinaldo 35 years ago in Pittsburgh. I was a critical care fellow, and Rinaldo was interviewing to join the program. Our program director, Ake Grenvik, asked me to take Rinaldo to lunch, where I was immediately impressed with Rinaldo’s certainty and conviction. He was so sure of what he wanted, and so focused in his questions about the program, that I felt quite thoughtless and indecisive by comparison regarding my own career path. I also wondered if he was perhaps a bit overly zealous. The following year, however, when Rinaldo began working in Pittsburgh, I quickly realised that, although he always argued points to death, he hoped for an equally vigorous counterargument. The better the debate, the bigger his smile, if not an outright guffaw. Crucially, for all the energy with which he argued, he was always ready and open to believe that the opposing argument could also be right. This was a remarkable trait, to bring all his energy to an idea and yet be ready in an instant to switch with humility and grace to an alternative, all in the name of simply trying to find something closer to the truth.

As Rinaldo was nearing the end of his fellowship and getting ready to return to Australia, I remember wonderful discussions about two prevailing concepts regarding acute renal injury. First, why were we taught that sepsis-induced acute renal injury was due to loss of renal blood flow? Second, why did we give the so-called “renal-dose dopamine” all the time? The first question simply required large animal models of sepsis, with flow probes on the renal artery. Sure enough, working in Michael Pinsky’s large-animal lab, Rinaldo verified that renal blood flow persisted in sepsis and could even be boosted with norepinephrine.^2^ Why we had all accepted the prevailing dogma when the answer was readily available was unclear but a source of wry amusement for Rinaldo. It was also not lost on Rinaldo that this work inched us forward a little but hardly solved acute kidney injury!

The second question, whether low-dose dopamine protected the kidney during critical illness, was trickier to answer as it would require a clinical trial, yet Rinaldo’s research experience was essentially just in the lab; he had never conducted a trial. Nevertheless, he figured he could run the trial in a bunch of Australian ICUs, also a first, and do so largely by the force of personality and persuasion. When he asked me about sample size and power calculations, I was quite skeptical about the whole venture, but I liked his enthusiasm. Of course, the rest was history: the finding of no benefit was published in the *Lancet*, leading quickly to an abandonment of another piece of dogma, and the Australian and New Zealand Intensive Care Society Clinical Trials Group was launched.^3^

Many years later, I remember sitting with Rinaldo on a sidewalk in Melbourne, feet pointing into the road, drinking a cup of coffee and discussing whether we could verify the benefits of early goal-directed therapy. I had just received National Institutes of Health (NIH) funding for Protocolized Care of Early Septic Shock (ProCESS) and wondered if he could submit an Australian version of the same trial to the 10.13039/501100000925National Health and Medical Research Council. If he was successful, we could then join forces for one huge international evaluation. It took about 15 min to decide we should do it. I wanted to show early goal-directed therapy would work; Rinaldo was sure it would not work but loved the idea that we should try to find out one way or the other! Again, Rinaldo galvanised his colleagues to get involved in a project that could revolutionise sepsis care, or not ...

Rinaldo also emailed me countless times about papers he thought would be great for *JAMA*. Every time, he would be spectacularly positive about the study, detailing how important the question was, how thoughtfully he and his coauthors had addressed the question, and why the findings would be so valuable for *JAMA’*s readership. I would often send these papers out for review, and we published several over years, but I think the more interesting story was about the many times we rejected his papers. Despite his prior rigorous defence, he was always incredibly thoughtful and gracious in response to our counterpoint. Just as when we were in Pittsburgh decades earlier, he would always argue the case with vigour and then always be ready to accept another position.

Perhaps the greatest scientific mind of the twentieth century, Richard Feynman, was quoted as saying “the first law of science is to never fool anyone; the second law is that the easiest person to fool is yourself”. Rinaldo’s ability to dive fearlessly and with gusto into any new scientific project and yet always be ready to say “ok, that didn't work” showed he lived by Feynman’s credo. He had a mind truly built for scientific discovery: boundless zeal matched by deep humility and self-reflection. In addition to remembering Rinaldo for the many milestones he achieved on his journey, we should also remember the way by which he walked his path.

## Disclosures

There are none to declare.

## Funding

This research did not receive any specific grant from funding agencies in the public, commercial, or not-for-profit sectors.

## Conflict of interest

The authors declare that they have no known competing financial interests or personal relationships that could have appeared to influence the work reported in this paper.
